# Long-term outcome of bone marrow transplantation in NIK deficiency: non-redundant role of non-canonical NF-κB signaling in thymic reconstitution and secondary lymphoid organ development

**DOI:** 10.3389/fimmu.2025.1682642

**Published:** 2025-11-07

**Authors:** Sevgi Köstel Bal, Şule Haskoloğlu, Bernhard Ransmayr, Selin Sevinç, Candan İslamoğlu, Kübra Baskın, Nazlı Deveci, Berna Savaş, Suat Fitoz, Alphan Küpesiz, Tanıl Kendirli, Kaan Boztug, Figen Doğu, Kamile Aydan İkincioğulları

**Affiliations:** 1 Department of Pediatric Allergy and Immunology, Gülhane Research and Training Hospital, University of Health Sciences, Ankara, Türkiye; 2 Department of Pediatric Immunology and Allergy, Ankara University School of Medicine, Ankara, Türkiye; 3 St Anna Children’s Cancer Research Institute (CCRI), Vienna, Austria; 4 CeMM Research Center for Molecular Medicine of the Austrian Academy of Sciences, Vienna, Austria; 5 Department of Pediatric Allergy and Immunology, Gazi University School of Medicine, Ankara, Türkiye; 6 Etlik City Hospital Department of Pediatric Allergy and Immunology, University of Health Sciences, Ankara, Türkiye; 7 Department of Pathology, Ankara University School of Medicine, Ankara, Türkiye; 8 Division of Radiology, Ankara University School of Medicine, Ankara, Türkiye; 9 Department of Pediatric Hematology, Akdeniz University Faculty of Medicine, Antalya, Türkiye; 10 Department of Pediatric Intensive Care, Ankara University School of Medicine, Ankara, Türkiye; 11 Department of Pediatrics and Adolescent Medicine, Medical University of Vienna, Vienna, Austria

**Keywords:** combined immunodeficiency, non-canonical NFKB signaling, hematopoietic stem cell transplantation, central tolerance, NIK deficiency

## Abstract

**Introduction:**

Biallelic mutations in *MAP3K14*, encoding NF-κB-inducing kinase (NIK), disrupt non-canonical NF-κB signaling and lead to a rare inborn error of immunity marked by impaired lymphoid organ development, defective lymphocyte maturation, and susceptibility to recurrent infections. Hematopoietic stem cell transplantation (HSCT) has been considered a curative approach, yet its long-term efficacy remains unclear.

**Method:**

We report long-term outcomes of two patients with genetically confirmed NIK deficiency who underwent HSCT.

**Results:**

Both patients achieved full donor chimerism and early T-cell reconstitution with normalized CD3^+^, CD4^+^, and CD8^+^ counts and naïve T-cell subsets. However, memory T-cell differentiation remained impaired, with persistently reduced central memory T cells and circulating T follicular helper cells. Immune dysregulation emerged years after HSCT, with one patient developing seropositive arthritis and the other exhibiting autoimmune hepatitis. Thymic dysfunction was suspected as an underlying contributor to impaired central tolerance in this pathology. Similarly, B-cell reconstitution was incomplete, characterized by persistent hypogammaglobulinemia and a marked deficiency in class-switched memory B cells, despite donor-derived chimerism. Lymphoscintigraphy confirmed absence of lymph nodes. Both patients suffered from recurrent, severe infections and ultimately died of infection-related complications. Our findings indicate that HSCT alone is insufficient to fully correct the immune disorder in *MAP3K14* deficiency, likely due to non-hematopoietic defects in lymph node stromal structures and thymic central tolerance.

**Discussion:**

These results highlight the importance of long-term immunologic monitoring, including assessments for immune dysregulation and anti-cytokine autoantibodies. Future therapies should consider adjunct strategies such as thymic regeneration or targeted immune modulation to address the underlying architectural defects in this disorder.

## 1 Introduction

The maintenance of immune homeostasis within lymphoid organs requires precise spatial regulation, ensuring coordinated interactions between lymphocytes and antigen-presenting cells within a well-structured stromal network (1–4). The nuclear factor kappa-light-chain-enhancer of activated B cells (NF-κB) signaling pathway has a pivotal role in both the development and the maintenance of the secondary lymphoid organs (SLOs) and related immune surveillance (5, 6). To date, numerous human immunodeficiencies have highlighted the essential contribution of NF-κB signaling to lymphoid immunity in terms of both the stromal and the lymphocytic component (7), however research and therapeutic approaches mainly focused on the lymphocytic compartment. The impact of the stromal defects to the clinical manifestations and related therapies remained underexplored.

The development of the stromal compartment of the SLOs relies mainly on the lymphotoxin activated non-canonical (or alternative) NF-κB signaling (8–10). The non-canonical pathway is activated gradually in response to a limited set of ligands, primarily from the tumor necrosis factor (TNF) superfamily, including lymphotoxin (LT), TNF-like weak inducer of apoptosis (TWEAK), B-cell activating factor (BAFF), CD40L, and receptor activator of nuclear factor kappa beta (RANKL) (6, 7). A key event in the non-canonical NF-κB pathway is the stabilization of NF-κB-inducing kinase (NIK), which subsequently activates IKK-α (11, 12). This activation leads to the phosphorylation and processing of NF-κB2/p100 into its transcriptionally active form p52, which forms a heterodimer with RelB. The resulting complex translocates into the nucleus and activates the expression of target genes (13, 14).

The impact of lymphotoxin signaling and non-canonical NF-κB pathway has been well established in murine models (4, 8, 9, 11, 13, 15, 16), and its clinical relevance has recently been underscored in human immunity by the identification of inherited deficiencies in LTβR and NIK (17, 18), both of which recapitulate the immune disorder including the complete lymph node aplasia observed in their murine counterparts. Despite extensive mechanistic insights into the NF-κB pathway, therapeutic options remain limited to lifelong intravenous immunoglobulin (IVIG) supplementation and/or HSCT. While LTβR-deficient patients generally fare well with IVIG replacement therapy, NIK-deficient patients exhibit severe, life-threatening combined immunodeficiency with increased susceptibility to bacterial, viral, and protozoan infections, often necessitating HSCT as a life-saving intervention (17, 18). Notably, transplantation experiments in mice have failed to fully correct the immunological defects (11, 15, 16, 19), raising concerns about the long-term outcomes of HSCT in NIK-deficient patients, despite initially promising clinical responses. Here, we present new data on B cell function in NIK deficiency and provide a long-term follow-up of two individuals (10 years and 4 years) who underwent HSCT. Our findings offer insights into alternative therapeutic strategies aimed at improving patient survival and immune function.

## 2 Method

### 2.1 Patients

Three individuals from a family affected with *MAP3K14* defect, who were under follow-up in Ankara University Department of Allergy and Immunology were retrospectively assessed in this study. Complete blood counts, immunoglobulin levels, lymphocyte subsets, lymphocyte activation responses and genetic aberrations were analyzed, as well as the clinical data and histopathological findings of biopsy specimens. Patients and families provided written informed consent in accordance with the Declaration of Helsinki. Study approval was granted by the institutional review boards, including Ethics Committees of Ankara University School of Medicine (I4-250-20) and Medical University of Vienna, Austria (1796/2018), Data from two patients (P1 and 2) have been previously published (17) with additional information including longer-term follow-up collected for this study.

### 2.2 Lymphocyte phenotyping and function

Peripheral blood was collected from healthy blood donors and patients with *MAP3K14* mutations. Proportions of CD3^+^, CD4^+^ T (CD3^+^CD4^+^), CD8^+^ T (CD3^+^CD8^+^), B cells (CD19 and CD20); naïve (N; CD45RA^+^CCR7^+^), central memory (T_CM_; CD45RA-CCR7^+^), effector memory (T_EM_; CD45RA^-^CCR7^-^), CD45RA^+^ revertant memory (T_EMRA_; CD45RA^+^CCR7^-^) cells (20); αβ (CD3^+^TCRαβ^+^) and γδ (CD3^+^TCRγδ^+^) T-cells; NK (CD3^-^CD56^+^); switched memory (CD19^+^IgM^-^27^+^IgD^-^), marginal zone (CD19^+^IgM^-^27^+^IgD^+^), naïve (CD19^+^IgM^+^27^-^IgD^+^), activated (CD19^+^CD38^Low^CD21^Low^) B-cell subsets (21) were determined by flow cytometry. Data was acquired on an LSRII SORP or LSR Fortessa (Becton Dickinson) and analyzed using FlowJo (Tree Star).

### 2.3 Co-culture for B cell activation and differentiation

The method was performed as previously reported (18). In brief, combinations of peripheral blood mononuclear cells (PBMCs), activated DCs and fibroblasts from each healthy individual were co-cultured with or without additional stimulation of the measles, mumps, and rubella vaccine (MMR, Merck Sharp und Dohme) and the NIK inhibitor B022 (MedChemExpress). PBMCs from healthy controls (HCs) were thawed, and CD14^+^ monocytes were isolated using CD14 MicroBeads (Miltenyi). To generate DCs, monocytes were cultured for six days in the presence of IL-4 (1000 IU/ml, Peprotech) and granulocyte-macrophage colony-stimulating factor (GM-CSF; 1000 IU/ml, Peprotech). On day six, DCs were stimulated with poly I:C (10 μg/ml, InvivoGen) for 24 hours. In parallel, CRISPR-Cas9–edited *B2M* knockout fibroblasts were seeded into 24-well transwells at 5000 cells per well. The following day, donor-matched PBMCs (300,000 cells per well) and DCs (30,000 cells per well) were added to the upper chamber, with or without the addition of 5 μl of MMR vaccine and 2μ/ml B022 per well. The lower chamber contained 1 ml of RPMI 1640 medium supplemented with 10% FCS. Cells were stimulated with 50 ng/ml BAFF (Peprotech) every other day. On day 11 of the co-culture, cells were harvested and analyzed for activation and differentiation using flow cytometry.

### 2.4 Statistical analysis

For single comparisons of independent groups, a Mann-Whitney test was performed. For multiple comparisons, a two-way analysis of variance (ANOVA) or multiple t-tests were applied. Analyses were performed using PRISM software (GraphPad Software Inc).

## 3 Results

### 3.1 Patient descriptions

In 2014, we identified two cousins from the same family harboring biallelic *MAP3K14* mutations. The clinical course prior to HSCT was described previously for P1 and P2 (17). P2 succumbed to sepsis on post-transplant day 6, thus her posttransplant follow-up will not be further discussed in this report. In 2016, P3 was born and identified to have the same homozygous mutation as his late sister P2 (**Table 1**; **Supplementary Figure 1**.). The detailed case descriptions following the transplantations of all three patients are provided in the supplementary section (**Supplementary File**). Here, a brief summary is provided for P1 and P3.

**Table 1:** Clinical features and posttransplant followup of patients with NIK deficiency.

Patients:	Patient 1	Patient 2	Patient 3
Age of diagnosis	9 years	3.5 years	10 months
Gender	Female	Female	Male
Mutation	*MAP3K14* c. C1694G, p. Pro565Arg	*MAP3K14* c. C1694G, p. Pro565Arg	*MAP3K14* c. C1694G, p. Pro565Arg
Clinical presentation prior to HSCT	CMV infection Cryptosporidium infestation Granulomatous hepatitis Tuberculosis osteomyelitis (BCG dissemination)	Lower respiratory tract infections Oral and esophageal candidiasis Chronic diarrhea Cholestasis	Parainfluenza pneumonia BCGitis
Donor type	MUD (10/10)	MFD (10/10)	MFD (10/10)
Conditioning regime	Busulfan (2.5 mg/kg) Fludarabin (30 mg/m^2^) ATG (10 mg/kg)	Treosulfan Fludarabine	Busulfan (16 mg/kg) (AUC: 100) Fludarabin (160 mg/m^2^)
Day of Engraftment	+13 (neutrophil)	N/A	+13 (neutrophil)
GvHD	Mild skin GvHD (grade 1-2) (+3 mo)	N/A	Skin GvHD grade 2 (+24 mo) Liver GvHD (see text)
Autoimmunity	Arthritis Anti-ds DNA + anti-SM + ANCA +	N/A	Ashy dermatosis Hepatitis (anti-LKM +)
Infections	Aspergillus fumigatus Actinomyces Pseudomonas aeruginosa SARS-Cov2 penumonia (x2) CMV sepsis	CMV sepsis	Crytopsporidium diarrhea Sphingobium Yanoikuaye, Burkholderia cephacia, Candida crusei Aspergillus flavus CMV sepsis
Post-HSCT follow up duration	10 years	N/A	4 years

AUC, area under the curve; CMV, cytomegalovirus; GvHD, Graft versus host disease; MUD, matched unrelated donor; MFD, matched family donor; N/A, not applicable.

**Figure 1:**
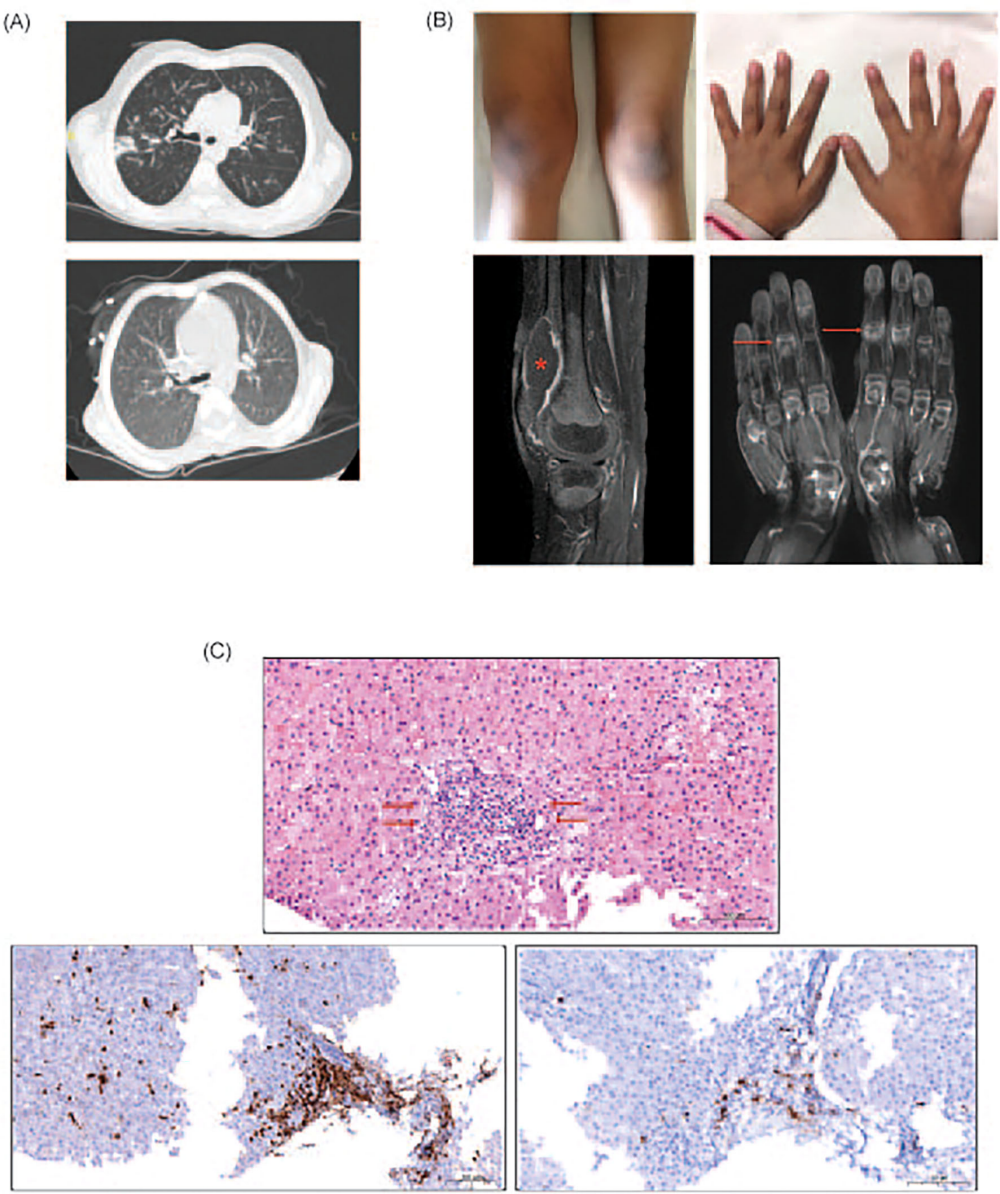
**(A)** Upper panel: Axial computer tomography (CT) image showing thickened bronchial walls and pneumonic infiltration in right upper lobe posterior segment. Image is from the episode where *Aspergillus fumigatus* was isolated from the bronchoalveolar lavage fluid of P1. Lower panel: Follow-up CT image demonstrating the complete resolution of the pneumonia. **(B)** Upper panel: Photos of arthritis on right knee and interphalangeal joints of P1 Lower panel: Contrast-enhanced MRI mages of both hands in coronal plan and right knee in sagittal plan showing synovial enhancements and inflammatory changes in interphalangeal and intercarpal joints (indicated with arrows). Please note the large effusion in the right knee (asterisk). **(C)** Upper panel: Hematoxylin eosin staining of the liver specimen from P3 showing the lymphocytic infiltration (indicated with arrow) at the periportal area in liver. Lower panel left: Immunohistochemistry staining of the liver specimen showing the CD3 positive lymphociytic infiltration at the periportal area Lower panel right: Immunohistochemistry staining of the liver specimen showing CD20 positive lymphocytic infiltration.

Patient 1 (P1) was a 9-year-old girl born to consanguineous parents, with a family history of a younger brother who died at the age of 2 years from suspected combined immunodeficiency. She was diagnosed with combined immunodeficiency based on clinical diagnosis and underwent allogeneic HSCT from a 10/10 matched unrelated donor following a reduced-toxicity conditioning regimen. Early post-transplant complications included mild skin graft-versus-host disease (GvHD), managed with cyclosporine, and cytomegalovirus (CMV) antigenemia, which responded to ganciclovir. Full donor chimerism was achieved, and immunosuppression and IVIG were discontinued after 10 months. However, at 24 months, she developed persistent CMV viremia and hypogammaglobulinemia, requiring irregular IVIG replacement. Bronchiectasis and pneumonitis (**Figure 1A**) due to *Pseudomonas aeruginosa* and CMV were later diagnosed, with CMV detected in bronchoalveolar lavage and resistant to ganciclovir but responsive to foscarnet. At 42 months, she developed seropositive inflammatory arthritis involving multiple joints (**Figure 1B**), resistant to non-steroidal anti-inflammatory drugs (NSAIDs) and steroids, but partially controlled with methotrexate. 2 doses of rituximab were administered and anti-BAFF treatment was planned, but she ultimately succumbed to Aspergillus pneumonia and myocarditis with malignant arrhythmia, despite extracorporeal membrane oxygenation (ECMO) support.

Patient 3 (P3), the younger brother of P2, presented in infancy with recurrent respiratory infections, candidiasis, and BCGitis. He shared the same *MAP3K14* mutation and immunophenotype as his late sister. At 11 months, he underwent HSCT from his HLA-identical mother following myeloablative conditioning. Engraftment was successful, and he was discharged on day 90 with complete donor chimerism. In the first two years, he had Cryptosporidium-related diarrhea and CMV viremia, but otherwise had a stable follow-up. Despite complete donor chimerism, IgG levels and switched memory B cells remained low, requiring ongoing IVIG. At 24 months, he developed ashy dermatosis and later cholestatic hepatitis with positive anti-LKM1 antibodies. The biopsy for skin and liver showed GvHD-like pathology findings. Immunosuppression including steroids, rapamycin, mycophenolate mofetil and tacrolimus partially controlled bilirubin levels. He experienced multiple pneumonias with opportunistic pathogens including *Burkholderia cepacia* and *Aspergillus flavus*. At post-transplant month 36, he was hospitalized with dysphagia and gastrointestinal bleeding, and despite treatment, died of septic shock. Post-mortem findings revealed CMV in the gastrointestinal tract.

### 3.2 HSCT was ineffective in rescuing memory T cell formation despite complete donor chimerism

To control the severe infections, HSCT was carried out in an emergency setting for P1 and P2 without knowing the genetic mutation. Due to hepatic dysfunction, a reduced-intensity conditioning regime was preferred in these patients. P2 succumbed to sepsis on post-transplant day 6. P3 received the genetic diagnosis before developing major infections, while P1 was already in the third-year post-transplant. Considering the complicated follow-up of his cousin, a myeloablative regimen was preferred for a stable graft (**Table 1**).

To assess the engraftment process and the restoration of lymphocytic function, we conducted routine chimerism and flow cytometric analyses alongside close clinical follow-up. By the third month post-transplant, both patients (P1 and P3) achieved complete donor chimerism (>90% donor cells in whole blood), and their T-cell subpopulations were comparable to those of age-matched healthy controls. Percentage distributions of CD3, CD4 and CD8 were normal in both patients. Naïve T cell proportions were higher in both CD4 and CD8 T-cell populations, with proportions of recent thymic emigrants (RTE) being normal, whereas CD4^+^CD45RO^+^ memory frequencies remained lower than the age-matched controls throughout the follow-up (**Table 2A and 2B**).

Despite initial immune reconstitution, both patients experienced recurrent infectious complications related to a T-cell defect. During the first year post-transplant, both developed viremia due to CMV reactivation. In subsequent years *Pneumocystis jirovecii* pneumonia, aspergillosis, and CMV pneumonitis (P1); *Cryptosporidium parvum* diarrhea, pneumonitis due to *Aspergillus flavus*, *Candida krusei* and *Sphingobium yanoikuaye* (P3) were documented in the patients. The chimerisms on T cells were above 90% in both patients throughout these infections. We therefore assessed the lymphocyte functions via T-cell activation responses measuring CD25 and CD69 upregulation following CD3/CD28 stimulation. The activation responses during the initial two years were normal whereas they gradually declined to approximately 40%. (**Table 2A**).

**Table 2A:** Immunereconstitution following the HSCT of P1.

	Pretransplantation no % sign	+6 months (2014)	+2 years (2015)	+3 years (2016)	4 years (2017)	5 years (2018)	6 years (2019)	8 years (2021)	9 years (2022)
CD3 + 16-56-(58-82%)(%)	93	65	69	81	91	86	88	94	94
CD3-16 + 56+(8-30%)(%)	1	5	4	1.2	3	2	3	3	2
CD3+CD4+(27-57%)(%)	76	25	40	52	58	57	53	53	69
CD3+CD8+(19-38%)(%)	19	50	41	28	33	35	41	41	29
CD4+CD45RO+(10-42%)(%)	26	22		6	15	11	20	7	11
CD4+CD45RA+(16-40%)(%)	75	14		52	74	75	41	43	56
CD19+(10-30%)(%)	3	20	10	7	3	3	3	0.4	0.6
CD20+(9-28%)(%)	4	20	10	7	3	3	2	0.3	0.5
CD45+CD4+CD31+ (RTE)		17	40	75	54	68	67	62	68
Immunoglobulin A(mg/dl) Immunoglobulin G(mg/dl) Immunoglobulin M(mg/dl)	24.9 (60-220) 320 (600-1300) 205 (40-160)	<6 800(ivig) 120	<6 336 167	<6 893 (ivig) 200					
Activation responses CD4+CD25+(%) (46-89%) CD4+CD69+(%) (50-76%)			98 98	56 49	40 37	69 63	43 42	61 60	73 72
B cell subsets CD19+IgM-CD27+IgD- (SM) (6.5 - 29.2%) CD19+IgM+CD27+IgD+ (MZ) (7.2 - 30.8) CD19+IgM+CD27-IgD+ (NB) CD19+CD38lowCD21low (AB) (1.1-6.9%)			0.1 94 5 6	0.1 98 1.7 8				0.1 94 5 5	
T cell subsets CD4+CD45RA+CCR7+ (naïve) (28.5-44%) CD4+CD45RA-CCR7- (EM) (10.6-34.2%) CD4+CD45RA-CCR7+ (CM) (15.9-34.6%) CD4+CD45RA+CCR7-(EMRA) (4.5-43.6)								83 10 2.5 3.9	78 8 13 0.95
CD4+CD45RA-CXCR5+(TFH) (5-15%)								2	
CD8+CD45RA+CCR7+(naïve) (5.5-39.7%) CD8+CD45RA-CCR7-(EM) (17-42.4%) CD8+CD45RA-CCR7+(CM) (1.5-3.9%) CD8+CD45RA+CCR7- (EMRA) (19.5-52.5%)								31 10 2 37	47 26 12 15
Chimerism(%)		87	95			95		90	

The relatively normal activation responses in a setting of repetitive infections prompted us to analyze T lymphocyte subpopulations, classified into 4 distinct populations: naïve (CD45RA^+^CCR7^+^); T_CM_ (CD45RA^-^CCR7^+^); T_EM_ (CD45RA^-^CCR7^-^) and T_EMRA_ (CD45RA^+^CCR7^-^) (20). Under physiological conditions, central memory (TCM) cells predominate within the CD4^+^ compartment. In our patients, proportions of naïve CD4^+^ T cells were increased, accompanied by a reduction in CD4^+^ TCM cells, a shift that was more pronounced in P1 and milder in P3, suggesting partial preservation of memory differentiation in the latter. (**Tables 2A**, **B**). Circulating T follicular helper cell frequencies were also reduced in P1. The CD8^+^ T-cell compartment showed relatively preserved distributions of naïve and memory subsets, consistent with the expected TEM predominance and without a consistent shift toward naïve enrichment (**Tables 2A**, **B**). Collectively, these findings indicate that the impairment in memory differentiation was more evident within the CD4^+^ lineage, which physiologically depends more heavily on secondary lymphoid organ integrity for effective TCM generation (22).

**Table 2B:** Immunereconstitution following the HSCT of P3.

	Pretransplantation	+3 months	+6 months	+9 months	+1 year	+15 months	+2 years	+3 years	+4 years
CD3 + 16-56-(%) (55-79%)	96	59	69	70	72	76	77	73	81
CD3-16 + 56+(%) (5-28%)	2	7	4	4	3	4	3	3	1
CD3+CD4+(%) (26-49%)	61	16	35	32	36	36	41	51	51
CD3+CD8+(%) (9-35%)	33	40	31	35	37	37	35	35	35
CD4+CD45RO+(%) (8-42%)	8	3	13	5	8	8	7	10	20
CD4+CD45RA+(%) (20-41%)	52	15	22	27	32	32	39	32	38
CD19+ (11-31)(%)	0.5	22	22	15	10	10	11	14	7
CD20+(%) (11-29%)	0.2	22	22	15	10	10	11	14	7
CD45+CD4+CD31+ (RTE)	74	67	50	75	74	74	83	67	
Activation responses CD3+CD25+(%) (46-89%) CD4+CD69+(%) (50-76%)	70% 66%	70% 65%	91 91	67 62	61 61	77 75	48 49	42 45	48 47
Immunoglobulin A(mg/dl) Immunoglobulin G(mg/dl) Immunoglobulin M(mg/dl)	<6 (57-282) 420 (745-1804) 102 (78-261)	<6 1080(ivig) 79	<6 621 69	<6 291 69	<6 161 66	<6 1020 (ivig) 80			
B cell subsets CD19+IgM-CD27+IgD- (SM) (6.5 - 29.2%) CD19+IgM+CD27+IgD+ (MZ) (7.2 - 30.8%) CD19+IgM+CD27-IgD+ (NB) CD19+CD38lowCD21low (AB) (1.1-6.9%)		35 1,7 62 28	3 1,7 93 2,6			10 2,1 87 1,2		0.1 6 93 8	1 5 85 4
T cell subsets CD4+CD45RA+CCR7+ (naïve) (28.5-44%) CD4+CD45RA-CCR7- (EM) (10.6-34.2%) CD4+CD45RA-CCR7+ (CM) (18.9-34.6%) CD4+CD45RA+CCR7-(EMRA) (4.5-43.6)								74 6 20 0.3	52 23 20 4
CD8+CD45RA+CCR7+(naïve) (5.5-39.7%) CD8+CD45RA-CCR7-(EM) (17-42.4%) CD8+CD45RA-CCR7+(CM) (1.5-3.9%) CD8+CD45RA+CCR7- (EMRA) (19.5-52.5%)								68 9 21 2	77 12 8 4
CD4+CD45RA-CXCR5+ (TFH) (5-15%)								4	
Chimerism(%)		99	84	90	98 (B cell 99%)	96		90	

AB, Activated B cells; CM, Central memory; EM, Effector memory; EMRA, effector memory cells re-expressing CD45RA; MZ, Marginal zone; NB, Naive B cells; RTE, Recent thymic emigrants; SM, Switched memory; TFH, T follicular helper cells.

Alymphoplasia (*aly/aly*) mice, which carry biallelic mutations in the kinase domain of NIK, are considered the prototype model for NIK deficiency (11, 13, 23, 24). Similar to the findings in these mice, our patients exhibited impaired cytotoxic T cell function following bone marrow transplantation. In the murine model, this dysfunction was attributed to the absence of lymph nodes and defective antigen presentation, despite transplantation with wild-type bone marrow (11). Together, these findings suggest a failure in memory T cell generation after transplantation.

### 3.3 Immunedysregulatory complications following HSCT

Previous studies have established that components of the non-canonical NF-κB signaling axis—namely NIK, IKK-α, and RELB—are indispensable for the functional maturation of medullary thymic epithelial cells (mTECs), which orchestrate central tolerance via ectopic expression of tissue-restricted antigens and the subsequent clonal deletion of autoreactive thymocytes (25–27). In light of this, and considering that our therapeutic strategy employed HSCT without concomitant thymic grafting, longitudinal surveillance for autoimmune phenomena was prioritized.

Autoimmune manifestations were not observed in the initial two years post-HSCT, mirroring the latency commonly reported for immune reconstitution-related complications. However, in the fourth post-transplant year, patient P1 developed seropositive oligoarticular arthritis (**Figure 1B**), accompanied by detectable anti-SSA and anti-SSM autoantibodies, despite persistent hypogammaglobulinemia and a paucity of class-switched memory B cells. The inflammatory arthritis was recalcitrant to conventional immunomodulators including methotrexate and rituximab. Initiation of anti-BAFF therapy was under consideration when the patient succumbed to septicemia. Patient P3 manifested signs of immune dysregulation at 24 months post-HSCT, initially presenting with ashy dermatosis followed by biochemical and serological evidence of hepatitis with anti-LKM1 autoantibodies. Histopathological evaluation of liver tissue could not definitively distinguish autoimmune hepatitis from GvHD. The hepatic involvement was refractory to multiple immunosuppressive regimens, including systemic corticosteroids, mesenchymal stromal cell infusions, mycophenolate mofetil, rapamycin, and tacrolimus. Retrospective re-evaluation of liver biopsy showed T and B cell infiltration in the periportal area (**Figure 1C**), similar to the pathology observed in patients with LTβR deficiency (18) and murine models of NIK deficiency (19). Collectively, the autoimmune pathology observed in these individuals phenocopies the autoimmunity seen in murine models deficient in NIK or LTβR signaling (8, 9, 13, 15, 24), further supporting the indispensable role of NIK within mTECs for the establishment of central tolerance in humans (28).

### 3.4 B cell compartment remained dysfunctional following the transplants despite complete donor chimerism

**Figure 2:**
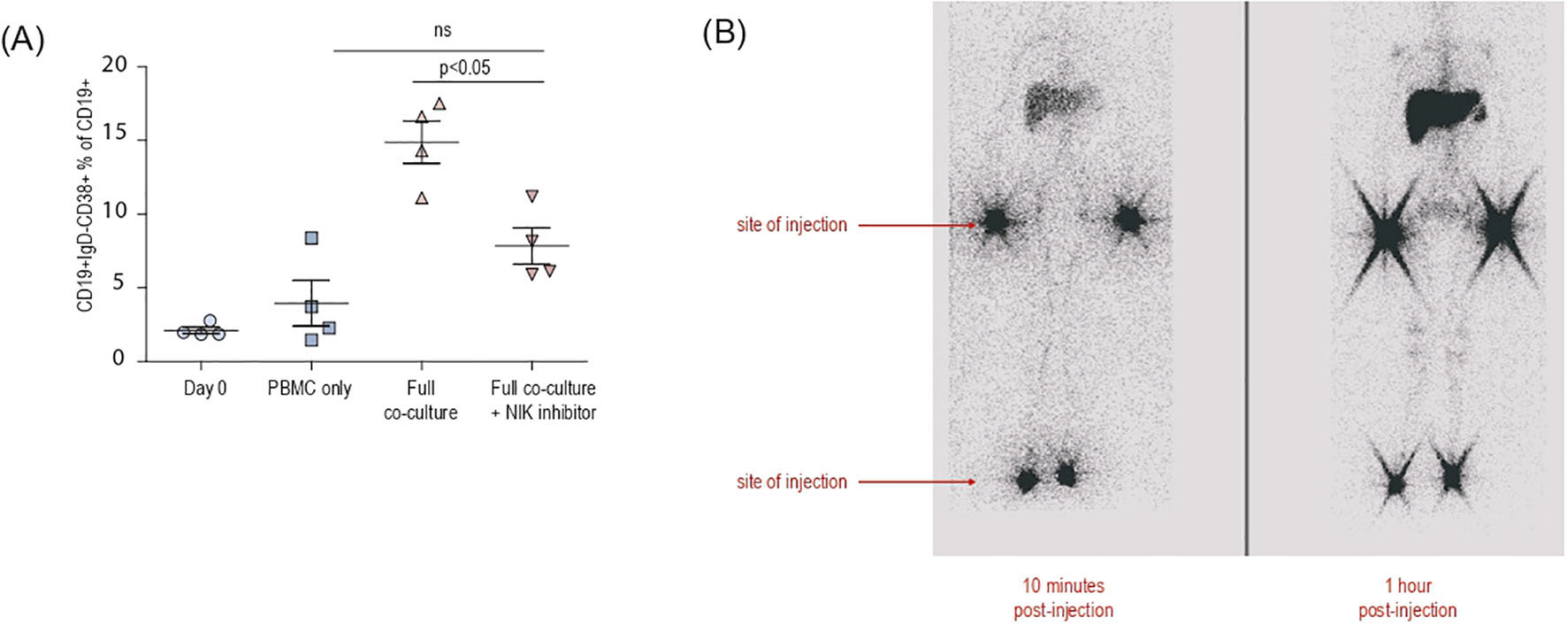
**(A)** Graph showing the percentage of the differentiation of plasmablast (CD19+IgD-CD38+) fraction within the CD19+ population. Combinations of peripheral blood mononuclear cells, activated dendritic cells and fibroblasts from each healthy individual donor were co-cultured with or without 2μ/ml B022 (NIK inhibitor) per well. **(B)** Lymphoscintigraphy of patient 1 following the HSCT at posttransplant year 4 showing the absence of lymph node structures.

Studies in the alymphoplasia (*aly/aly*) and Nik-knockout mice described B cell deficiency due to disorganized lymph nodes, Peyer’s patches and splenic architecture, accompanied by B cell lymphopenia and low serum Ig levels due to compromised class switch recombination (CSR) and somatic hypermutation (SHM) (8, 9, 11, 13, 24, 29). All three patients presented with profound B cell lymphopenia, low IgG and IgA accompanied with elevated IgM levels indicating a class switch defect. To better understand the impact of NIK deficiency on B cell activation and differentiation beyond the developmental defect on SLO development, we utilized a recently established co-culture system (18) that enables investigation of B cell interactions and responses *in vitro*. Co-culturing stromal cells, activated dendritic cells (DCs) and PBMCs results in the activation of B cells and stimulates them towards differentiation into plasmablasts (**Figure 2A**). Treatment with the NIK inhibitor B022 significantly abrogated B cell differentiation highlighting the importance of functional NIK signaling for B cell interaction and differentiation. (**Figure 2A**).

In *aly/aly* mice, transplantation of wild-type bone marrow fails to restore the development of peripheral lymphoid organs, such as lymph nodes and Peyer’s patches, and the splenic architecture remains disrupted (11). In our study, lymphatic scintigraphy was not performed prior to HSCT, and ethical constraints precluded the collection of tissue biopsies to directly assess SLO development in patients (17). Nevertheless, immunophenotypic reconstitution of the B cell compartment was evident by the first post-transplant month in both patients, with peripheral B cell counts reaching age-appropriate reference levels (**Tables 2A**, **B**; **Supplementary Figure 2**). IVIG replacement therapy was discontinued after nine months; however, patients continued to exhibit hypogammaglobulinemia characterized by persistently elevated IgM, consistent with an underlying defect in immunoglobulin CSR.

Despite full donor-derived B cell chimerism, flow cytometric analysis revealed a markedly reduced frequency of class-switched memory B cells within the total B cell pool (**Table 2**), prompting further investigation into the integrity of secondary lymphoid tissue. No palpable lymph nodes were identified on physical examination or visualized by ultrasonography. Subsequently, lymphoscintigraphy was performed and confirmed the complete absence of functional lymph nodes (**Figure 2B**). In addition, patient P1 underwent a lung biopsy to elucidate the etiology of post-transplant pneumonitis. During the procedure, tissue structures adjacent to the hilar region—initially presumed to be lymph nodes based on thoracic CT imaging—were excised and examined histologically. However, no organized lymphoid architecture was identified.

These findings collectively demonstrate that biallelic NIK mutations in humans recapitulate the immunological phenotype observed in *aly/aly* mice, including the failure of lymphoid tissue development and the non-responsiveness of the lymphoid stromal compartment to hematopoietic stem cell correction.

## 4 Discussion

Combined immunodeficiency due to biallelic germline mutations in *MAP3K14*, which encodes NF-κB-inducing kinase (NIK), was first described by our group in 2014 (17). The phenotype is characterized by increased susceptibility to recurrent bacterial, viral, and *Cryptosporidium* infections, along with impaired humoral immunity and persistent hypogammaglobulinemia. Although the essential role of NIK-mediated non-canonical NF-κB signaling in B cell maturation and SLO development was well recognized, its impact on T cell immunity had remained less well-defined. Since then, five additional patients have been reported, further highlighting the critical role of NIK in T cell biology—particularly in immunity against mycobacterial infections—and expanding the clinical spectrum associated with NIK deficiency (17, 30, 31). While our understanding of the pathobiology and immunological consequences of impaired NF-κB signaling has significantly improved, treatment options remain limited to HSCT, with long-term outcome data being scarce.

Here, we report the first long-term post-HSCT outcomes of two patients with NIK deficiency who underwent HSCT (P1: 10 years; P3: 4 years), providing insight into the clinical trajectory and immunologic reconstitution in the post-transplant setting. In both cases, HSCT—regardless of conditioning regimen or donor type—initially led to partial correction of the immunodeficiency and reduced susceptibility to infections. However, this benefit was not sustained. Several factors may explain the subsequent decline in immune function. Notably, lymphoscintigraphy confirmed the absence of lymph nodes suggesting that critical antigen presentation processes and the development of memory lymphocyte subsets were not re-established. This was reflected in persistently low frequencies of central memory and follicular helper T cells. Furthermore, B cell responses—particularly germinal center formation and CSR—remained impaired, necessitating continuous IVIG supplementation. An additional consideration is the possible contribution of anti–type I interferon autoantibodies, which we were unable to evaluate in our patients. Recent work by Le Voyer et al. has shown that following HSCT, patients with RELB defects may develop anti–type I interferon autoantibodies, predisposing them to life-threatening viral infections despite apparent immune reconstitution (32). While we cannot confirm this mechanism in our patients, the recurrence of infections raises the possibility of similar immune dysregulation. This warrants further exploration in future cases of NIK deficiency.

These findings underscore the essential role of NIK not only in hematopoietic cells but also in maintaining the structural and functional integrity of lymphoid architecture. In contrast to the study by Farhat et al., which reported partial reconstitution of the B cell compartment following HSCT (30), we were unable to corroborate such recovery in our cohort. The essential function of LTβR and its downstream non-canonical NF-κB signaling pathway for the formation of lymph nodes has been well-established in murine models. Notably, bone marrow transplantation has been shown to be insufficient for restoring lymph node development in *aly/aly* and LTβR knockout mice (11, 15, 16, 19). The impact of defective non-canonical NF-κB signaling appears to vary depending on the stage of embryonic development. In our patients, who harbor mutations within the catalytic domain of NIK, HSCT alone failed to restore B cell function—likely due to irreversible defects in early lymphoid organogenesis and impaired B cell trafficking. Whether this reflects a mutation-specific effect related to disruptions in the catalytic domain of NIK remains to be clarified through studies involving larger cohorts.

Previous studies have demonstrated that outcomes following HSCT in patients with NF-κB pathway defects are highly variable. These outcomes are influenced by several factors, including age at transplantation, underlying comorbidities, conditioning regimens, donor source, and post-transplant care (33). Reported post-transplant complications in this patient population include hepatotoxicity, poor engraftment, incomplete immune reconstitution, and the persistence or emergence of autoimmune manifestations (14, 32, 33). To date, only a limited number of patients with defects in the non-canonical NF-κB pathway—specifically involving NIK (4 patients), RELB (3 patients), and NFKB2 (1 patient)—have undergone HSCT (14, 30, 32, 33). In the single reported case of NFKB2 deficiency treated with HSCT, the patient succumbed to early post-transplant complications without evidence of durable immune reconstitution (14). Similarly, among the three RELB-deficient patients who underwent HSCT, two exhibited only partial immune recovery, accompanied by autoimmune manifestations, underscoring the limited capacity of HSCT to correct the non-hematopoietic components of the NF-κB pathway (30, 32). While engraftment has been successfully achieved in several cases, follow-up durations are generally less than five years, limiting insight into the long-term safety and efficacy of this therapeutic approach.

Non-canonical NF-κB signaling, particularly downstream of RANK, CD40, and LTβR, plays a critical role in shaping the thymic microenvironment and promoting central tolerance (34). Importantly, because mTECs—which are essential for T cell selection and the establishment of central tolerance—are non-hematopoietic in origin (15, 16, 19, 25, 26), HSCT cannot correct intrinsic defects in mTECs. Consequently, autoimmunity may persist or even newly arise following transplantation, as observed both in our patients and other non-canonical NF-κB defects. Post-transplant autoimmune manifestations have included hepatitis, arthritis, autoimmune cytopenias, and thyroiditis (14, 30, 32, 33). Considering the recently reported murine models, the severity of these conditions may be linked to the extent of thymic dysfunction and impaired regulatory T cell (Treg) development (35). Furthermore, as discussed earlier, even infectious complications could be secondary to thymic dysfunction—potentially through the development of anti-interferon autoantibodies (32). Although such autoantibodies were not assessed in our NIK-deficient cohort, the infections observed following transplantation raise concerns about a similar underlying mechanism, consistent with other inborn errors of immunity associated with anti-cytokine autoimmunity.

In addition to NIK, we have recently described the clinical phenotype associated with LTβR deficiency, which is also a key component for thymic development (18). LTβR signaling also supports the development and maintenance of thymic stromal cell subsets and promotes chemokine production, thereby contributing to broader thymic architecture and function. This differs substantially from AIRE deficiency (36), the prototype disease of thymic dysfunction—as LTβR-deficient patients predominantly present with B cell dysfunction in the absence of IFN-γ–driven chronic inflammation and autoimmunity. In these cases, IVIG replacement alone has been sufficient to manage clinical symptoms (18), and considering the clinical outcome of HSCT in NIK-deficient patients we have judged that HSCT would likely result in more harm than benefit.

Given these complex and multifactorial challenges, long-term immune monitoring is essential in this population. This should include regular screening for anti–type I IFN autoantibodies to identify patients at risk for delayed infectious complications. Assessment of thymic function and T cell repertoire diversity is also crucial to evaluate ongoing immune dysregulation. In selected cases, biomarker-driven immunomodulatory therapies—such as targeted B cell depletion—may help manage autoimmunity while preserving immune competence. Additionally, thymic regenerative strategies, including thymus transplantation or mTEC-targeted interventions (37–39), warrant further investigation as potential approaches to restore immune tolerance.

Overall, our findings highlight the complexity of immune reconstitution in non-canonical NF-κB pathway disorders. Despite initial clinical improvement following HSCT, persistent defects in lymphoid architecture and central tolerance mechanisms may limit long-term immune recovery. These observations underscore the need for precision medicine approaches—including personalized conditioning, immune monitoring, and organ-specific regenerative strategies—to improve long-term outcomes in this rare yet severe group of primary immunodeficiencies.

## Data Availability

The original contributions presented in the study are included in the article/**Supplementary Material**. Further inquiries can be directed to the corresponding author.
