# Supplementary material for: Long-term outcome of bone marrow transplantation in NIK deficiency: non-redundant role of non-canonical NF-κB signaling in thymic reconstitution and secondary lymphoid organ development
Source: Front Immunol. 2025 Nov 7;16:1682642. doi: 10.3389/fimmu.2025.1682642 (PMC12634528; doi:10.3389/fimmu.2025.1682642)
Supplement: Supplementary file 1 [file Supplementaryfile1.docx]

Supplementary Material

**Supplementary patient information**

**Supplementary figure 1. Pedigree indicating the patients**

**Supplementary Figure 2. Post-transplant kinetics of T- and B-cell reconstitution**

**Supplementary Table 1. List of Antibodies used in this study**

**References**

**Supplementary Patient Information**

Patient 1 (P1) was a 9-year-old girl born to consanguineous healthy parents (Supplementary Fig. 1a). The patient had a younger brother who died at the age of 2 years from suspected combined immunodeficiency. The clinical course prior to HSCT has been described previously(1). At the age of 9, she underwent allogeneic HSCT from a 10/10 HLA-matched unrelated donor, following a reduced toxicity conditioning consisting of busulfan (7.5 mg/kg), fludarabine (180 mg/m^2^), ATG (40 mg/kg). She developed mild skin GvHD which responded well to cyclosporin A. CMV antigenemia was detected through surveillance on the posttransplant day 40 which was controlled by gancyclovir. She achieved complete donor chimerisim and was discharged from the hospital. Both immunosuppression and intravenous immunoglobulin (IVIG) replacement were discontinued 10 months after transplant, and the post-transplant vaccinations were started. However, at +24 months she developed CMV viremia (peripheral blood CMV PCR: 1100 copies/µl) and hypogammaglobulinemia. IVIG replacement was reinitiated, but the episodes were irregular due to patient compliance. Subsequently, she developed a chronic productive cough where bronchiectasis with ground glass opacities were observedin a thorax CT. Bronchoscopy was performed and CMV PCR was detected as 45,000 copies/mL in bronchoalveolar lavage fluid. Additionally, sputum culture was positive for *Pseudomonas aeruginosa*. The CMV viremia was resistant to gancyclovir, but responded to foscarnet, and the *Pseudomonas* infection was controlled with meropenem. At 42 months post-transplant, she developed sterile monoarthritis of the knee (the synovial fluid culture was negative), which later involved both wrists and interphalangeal joints. Autoantibody testing was positive for anti-ds DNA, anti-Smith (anti-SM) and antineutrophil cytoplasmic antibodies (ANCA).. The arthritis was resistant to naproxen and corticosteroid joint injections, thus she was treated with methotrexate, which controlled progression of her symptoms. Despite strict home isolation, she developed three episodes of SARS-CoV-2-associated pneumonia. Thorax CT revealed also fungus ball and she was operated for the removal of it, during the operation hilar tissues which were suspected as the potential lymph nodes were resected. Pathological evaluation of these tissues just showed fibrotic adipose tissues without any lymph node structures. In light of the ongoing autoimmune manifestations and autoantibody production, treatment with rituximab was considered and 2 doses were administered. However, she ultimately succumbed to *Aspergillus sydowil* and *Stenotrophomonas maltophilia* pneumonia and myocarditis, complicated by malignant arrhythmia, despite extensive intensive care management including extracorporeal membrane oxygenation (ECMO) support..

Patient 2 (P2) was a first-degree cousin of P1 and was also born to consanguineous, healthy parents. She presented with recurrent lower respiratory tract infections, oral and oesophageal candidiasis and severe chronic diarrhea resistant to budesonide-mesalazine. She was tested positive for Cryptosporidium on one occasion later on she also developed cholestasis with deterioration of liver function. Her HLA-identical donor served as a donor for an allogeneic HSCT performed without conditioning at the age of 3. As no engraftment was observed after 50 days, a second transplant from the same donor was performed following treosulfan and fludarabine conditioning (Table 1). However, the patient succumbed to septic shock and multi-organ failure on day 6 following the second HSCT.

Patient 3 (P3) is the younger brother of P2. He presented with recurrent lower respiratory tract infections, candidiasis and BCGitis soon after birth. Immunophenotyping was similar to those of his late sister. (Table 2) Sanger sequencing confirmed the mutation in the *MAP3K14* gene. His mother, who was 10/10 HLA-identical, served as a donor for an allogeneic HSCT performed at the age of 11 months following a myeloablative conditioning regimen consisting of busulfan (16 mg/kg) and fludarabine (160 mg/m2). Methotrexate and cyclosporine A were used as prophylaxis for GvHD. Neutrophil and platelet engraftment occurred on post-transplant days 13 and 22, respectively. He was discharged from the hospital uneventfully on post-transplant day 90 having achieved complete donor chimerism. During the initial 2 years following the transplant, he had an uncomplicated clinical course, apart from two episodes of Crytopsporium-related diarrhea and CMV viremia. Despite complete donor chimerism on B cells, immunglobulin G levels and switched memory cells were lower than the age references. Ttherefore, IVIGreplacement had to be continued. At 24 months post-transplant he developed ashy dermatosis. The skin biopsy was compatible with grade I/II GvHD. Two months later he exhibited elevated liver enzymes progressing to cholestatic hepatitis. Autoantibodies were negative except for liver-kidney microsomal type 1 (LKM1) antibodies. The liver biopsy showed myelomonocytic and eosinophilic infiltration, consistent with GvHD. Rapamycin and mycofenolate mofetil were commenced to control the GvHD, but rapamycin had to be replaced with tacrolimus due to hyperlipidemia. Following the initiation of the immunsuppresive therapy, bilirubin levels stabilized but did not return to normal. He experienced multiple episodes of pneumonia, with *Sphingobium Yanoikuaye, Burkholderia cephacia, candida Crusei* and *Aspergillus* *flavus* isolated from bronchoalvelolar lavage fluid during different episodes. At 42 months post-transplant, he was hospitalized with dysphagia, endoscopy was planned. He developed contracted esophagial bleeding following the endoscopy and hospitalized in intensive care unit where he eventually succumbed to a septic shock. Post-mortem biopsies revealed CMV infiltration of the gastrointestinal tract (stomach, esophagus and duodenum).

:

**Supplementary Figures**

**Supplementary figure 1. Pedigree indicating the patients**

**
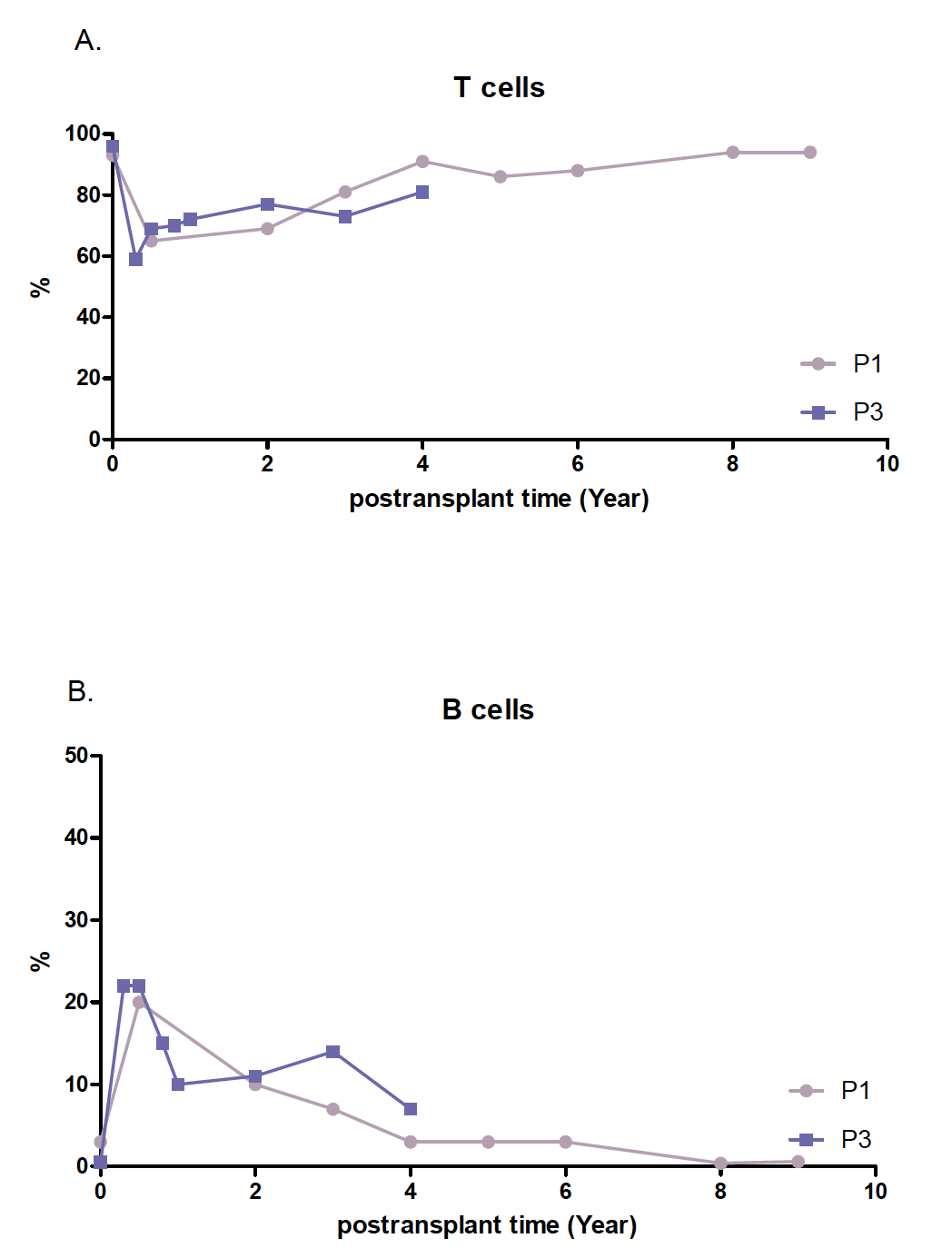
**

**Supplementary Figure 2. Post-transplant kinetics of T- and B-cell reconstitution** **A.** T cell percentage trend following transplantation **B**. B cell percentage trend following transplantation

**,**

**Supplementary Table 1. List of Antibodies used in this study**

| **Antibody-fluorophore** | **Clone** | **Manufacturer** |
| --- | --- | --- |
| CD19-PE-Cy7 | J3-119 | Beckman Coulter |
| CD19-BV510 | SJ25C1 | BD Biosciences |
| CD19-APC-Cy7 | SJ25C1 | BD Biosciences |
| CD19-PerCpCy5.5 | HIB19 | Invitrogen |
| CD38-BV711 | HIT2 | BD Biosciences |
| IgD-PE-TR | IA6-2 | BD Biosciences |
| CD45 KrO | J33 | Beckman Coulter |
| CD3 FITC | UCHT-1 | Beckman Coulter |
| CD3 PB | UCHT1 | Beckman Coulter |
| CD16+56 PE | 3G8+N901 | Beckman Coulter |
| CD4 ECD | SFCI12T4D11(T4) | Beckman Coulter |
| CD4 FITC | 13B8.2 | Beckman Coulter |
| CD8 APC-AF750 | B9.11 | Beckman Coulter |
| CD45RA PC7 | 2H4LDH11LD8 (2H4) | Beckman Coulter |
| CD45RO PE | UCHL1 | Beckman Coulter |
| HLA-DR PB | Immu-357 | Beckman Coulter |
| CD19 APC | J3-119 | Beckman Coulter |
| CD20 APC-AF750 | B9E9(HRC20) | Beckman Coulter |
| TCR gama/delta PC7 | IMMU510 | Beckman Coulter |
| HLA-ABC FITC | B9.12.1 | Beckman Coulter |
| CD31 PE | 1F11 | Beckman Coulter |
| CD197 (CCR-7) PE | G043H7 | Beckman Coulter |
| CD21 PE | BL13 | Beckman Coulter |
| CD25 PC7 | B1.49.9 | Beckman Coulter |
| CD27 PC5.5 | 1A4CD27 | Beckman Coulter |
| CD19 PC7 | J3-119 | Beckman Coulter |
| CD38 APC-AF750 | LS198-4-3 | Beckman Coulter |
| CD69 PC5 | TP1.55.3 | Beckman Coulter |

**References**

1. Willmann KL, Klaver S, Dogu F, Santos-Valente E, Garncarz W, Bilic I, et al. Biallelic loss-of-function mutation in NIK causes a primary immunodeficiency with multifaceted aberrant lymphoid immunity. Nature communications. 2014;5:5360.
